# Significance of Systemic Immune‐Inflammation and Inflammatory Response Indices in Vestibular Neuritis

**DOI:** 10.1111/coa.70140

**Published:** 2026-07-06

**Authors:** Tuğba Tulacı, Mustafa Utku Akbaş, Hasan Çanakçı, Tarık Yetişir, Kamil Gökçe Tulacı, Haşmet Yazıcı, Gulendam Kayıkçı, Omer Hizli

**Affiliations:** ^1^ Department of Otolaryngology, Faculty of Medicine Balikesir University Balikesir Turkiye; ^2^ Private Practice Balikesir Turkiye

**Keywords:** NLR, SII, SIRI, spontaneous nystagmus, systemic inflammatory markers, vestibular neuritis

## Abstract

**Introduction:**

This study investigated the relationship between vestibular neuritis and various systemic inflammatory indices—namely, neutrophil‐to‐lymphocyte ratio (NLR), platelet‐to‐lymphocyte ratio (PLR), monocyte‐to‐lymphocyte ratio (MLR), systemic immune‐inflammation index (SII), and systemic inflammation response index (SIRI) and the clinical severity of vestibular neuritis.

**Methods:**

A total of 79 individuals were included: 41 patients with vestibular neuritis and 38 healthy controls. Systemic inflammatory parameters were calculated from complete blood count results, and their associations with disease severity—classified as mild (≤ 4 days of nystagmus) or severe (> 4 days of nystagmus)—were analysed.

**Results:**

NLR, SII and SIRI were significantly higher in the severe vestibular neuritis group compared to controls (*p* = 0.032, *p* = 0.019 and *p* = 0.042, respectively), while the mild group showed no significant difference relative to either controls or the severe group. SII demonstrated the highest predictive value for severe vestibular neuritis (AUC = 0.66), showing superior discriminative performance compared with NLR and SIRI.

**Conclusion:**

Although the pathogenesis of vestibular neuritis remains unclear, the elevation of NLR, SII and SIRI in more severe cases suggests an inflammatory involvement. Their association with severe disease may support future research into whether these markers could help identify patients who are more likely to benefit from anti‐inflammatory treatment strategies. However, further prospective studies are needed to clarify the clinical relevance of these findings.

## Key Points

- NLR, SII and SIRI were elevated in severe vestibular neuritis.
- Severe disease was associated with increased systemic inflammatory burden.
- These findings support an inflammatory contribution to disease expression.
- Inflammatory markers were not correlated with vHIT‐based dysfunction severity.
- Further studies should explore whether patients with higher inflammatory burden represent a distinct subgroup with different treatment needs, including possible benefit from anti‐inflammatory therapy.

## 1 Introduction

Vestibular neuritis is a relatively common cause of dizziness, accounting for a significant proportion of acute peripheral vestibular disorders. It is recognised as one of the most frequent conditions following benign paroxysmal positional vertigo. The diagnosis is primarily based on the sudden onset of vertigo without accompanying symptoms such as tinnitus, idiopathic sudden sensorineural hearing loss or neurological findings. Infectious diseases, vascular compromise and autoimmune responses have been proposed to explain its aetiology, and these hypotheses suggest that inflammation plays a central role; however, the exact cause remains uncertain [1]. As a routine and accessible laboratory test, complete blood counts (CBCs) provide clinicians with indirect yet meaningful insight into both systemic physiological integrity and underlying inflammatory processes. Among the derived parameters, ratios such as platelet‐to‐lymphocyte (PLR) and neutrophil‐to‐lymphocyte (NLR) have recently emerged as practical reflections of systemic inflammatory burden and prothrombotic states [2]. Furthermore, several studies in the otorhinolaryngology field have reported increased PLR and NLR levels associated with idiopathic sudden sensorineural hearing loss and vestibular neuritis [3, 4, 5]. Recently, additional inflammatory indices such as the systemic immune‐inflammation index (SII), defined as platelet × neutrophil/lymphocyte, and the systemic inflammation response index (SIRI), calculated as neutrophil × monocyte/lymphocyte, have gained increasing attention. While SII has been investigated as a prognostic factor in malignancies [6, 7], emerging evidence also suggests a potential role for both SII and SIRI in various inflammatory and otorhinolaryngologic disorders, including idiopathic sudden sensorineural hearing loss and idiopathic facial paralysis [8, 9, 10]. These indices are believed to reflect the overall activity of the host inflammatory and immune status, thereby providing additional prognostic and diagnostic information.

### 1.1 Objectives

Given that vestibular neuritis shares an inflammation‐based aetiology like idiopathic sudden sensorineural hearing loss and idiopathic facial paralysis [11], we hypothesised that systemic inflammatory indices—namely NLR, PLR, MLR, SII and SIRI—are associated with the disease. Accordingly, this study aimed to evaluate the relationship between these inflammatory markers and the clinical severity of vestibular neuritis.

## 2 Materials and Methods

### 2.1 Study Design, Setting and Ethical Considerations

This retrospective study was approved by the Balikesir University Institutional Ethics Committee (IEC Number: 2023/112) and conducted in accordance with the Declaration of Helsinki and was reported in accordance with the STROBE guidelines.

### 2.2 Paticipants

Informed consent was obtained from all participants. A total of 79 individuals treated at our tertiary institution were included. Individuals with the following conditions were excluded: those with new or chronic infections; chronic inflammatory diseases; a history of acute hearing loss or tinnitus; persistent dizziness inconsistent with the acute course of vestibular neuritis; brainstem or cerebellar lesions on magnetic resonance imaging (MRI); any neurological deficits suggestive of central vestibular involvement; a history of Meniere's disease; ophthalmological disorders precluding reliable nystagmus evaluation; any malignancy; and all individuals under the age of 18.

A control group, matched for age and gender, was assembled from adult individuals (aged ≥ 18 years) who had previously undergone routine health check‐ups, including CBC tests, and had no documented history or symptoms of vertigo, hearing loss or tinnitus. Given the retrospective design of the study, additional imaging or audiological testing was not performed in this group.

The diagnosis of vestibular neuritis was established clinically based on the presence of acute, prolonged vertigo accompanied by spontaneous peripheral‐type nystagmus, the absence of auditory symptoms such as hearing loss or tinnitus, and the lack of neurological or other central signs. Pure‐tone audiometry was performed in all patients to confirm normal hearing thresholds and to exclude cochlear involvement. The video head impulse test (vHIT) was used to document unilateral vestibular hypofunction, and MRI was performed to exclude brainstem or cerebellar lesions indicative of central vestibular pathology. vHIT findings were classified topographically as superior, inferior or combined vestibular involvement. For exploratory analysis, the lowest canal gain recorded on vHIT was used as an indicator of vestibular dysfunction severity, and its relationship with inflammatory markers was assessed using Spearman correlation analysis.

Patients with vestibular neuritis underwent daily follow‐up including spontaneous nystagmus examinations and serial vHIT assessments. Cases were classified as mild when spontaneous nystagmus resolved within 4 days without hospitalisation, and as severe when hospitalisation was required or nystagmus persisted beyond 4 days, as described in a previous study [12].

CBCs from the initial visit were reviewed, and systemic inflammatory markers were calculated as follows:

- *NLR* = neutrophil count divided by lymphocyte count.
- *PLR* = platelet count divided by lymphocyte count.
- *MLR* = monocyte count divided by lymphocyte count.
- *SII* = (platelet count × neutrophil count) divided by lymphocyte count.
- *SIRI* = (neutrophil count × monocyte count) divided by lymphocyte count.

### 2.3 Main Outcome Measures

The systemic inflammatory markers were compared across mild vestibular neuritis, severe vestibular neuritis and control groups.

### 2.4 Statistical Analysis

All statistical analyses were conducted using non‐parametric methods due to the non‐normal distribution of the data, as verified by the Kolmogorov–Smirnov test (*p* < 0.05). Continuous variables were summarised as medians with corresponding minimum and maximum values. To compare differences among the three study groups, the Kruskal–Wallis test was applied. In cases where overall significance was observed, post hoc pairwise comparisons were performed and Bonferroni‐adjusted *p* values generated by the SPSS pairwise comparisons procedure were reported to account for multiple testing. Spearman correlation analysis was used to evaluate the relationship between inflammatory biomarkers and exploratory clinical parameters. For statistical analysis, SPSS 30.0 software for MacOs (SPSS Inc., Chicago, IL) was used. Statistical significance was defined as a *p* value < 0.05.

## 3 Results

### 3.1 Participant Characteristics

A total of 79 participants were enrolled, including 38 healthy controls and 41 individuals with vestibular neuritis. The control group comprised 38 participants [20 males and 18 females, median age: 45 (24–75) years], the mild vestibular neuritis group was composed of 17 patients [8 males and 9 females, median age: 54 (19–75) years], and the severe vestibular neuritis group included 24 participants [9 males and 15 females, median age: 49 (34–78) years]. The groups were comparable in terms of age and gender (*p* = 0.059 and *p* = 0.508, respectively) (Table 1).

**TABLE 1:** Demographic characteristics of the groups.

Variable	Control group (*n* = 38)	Mild vestibular neuritis (*n* = 17)	Severe vestibular neuritis (*n* = 24)	*p*
Age, median (min–max), years	45 (24–75)	54 (19–75)	49 (34–78)	0.059*
Sex, male/female	20/18	8/9	9/15	0.508^†^

*Note:* Demographic characteristics were compared across the three groups, with age analysed using the Kruskal–Wallis test and sex distribution assessed using the chi‐square test.

* Kruskal–Wallis test.

^†^ Chi‐square test.

### 3.2 Comparison of Inflammatory Markers Among the Groups

Kruskal–Wallis analysis revealed no statistically significant differences in PLR and MLR levels across the three groups (*p* = 0.092 and *p* = 0.203, respectively). However, statistically significant differences were found in NLR, SII and SIRI levels among the three groups (Table 2).

**TABLE 2:** Comparisons of blood parameters among three groups.

	Control group	Mild vestibular neuritis	Severe vestibular neuritis	*p* *
NLR	1.95 (0.9–4.21)	2.1 (1.42–7.61)	2.4 (1.09–9.62)	**0.023**
PLR	113.6 (71.7–205.7)	136.3 (81.4–305.4)	139.6 (61.1–317.3)	0.092
MLR	0.24 (0.15–0.43)	0.25 (0.105–0.578)	0.27 (0.142–0.625)	0.203
SII	464.2 (46.6–1227.8)	609 (366.4–2269.4)	642.1 (223.6–2782.1)	**0.017**
SIRI	0.99 (0.136–0.2756)	1.23 (0.49–5.33)	1.5 (0.47–4.81)	**0.036**

*Note:* Bold values indicate statistical significance (*p* < 0.05).

Abbreviations: MLR, Monocyte to lymphocyte ratio; NLR, Neutrophil to lymphocyte ratio; PLR, Platelet to lymphocyte ratio; SII, Systemic immune‐inflammation index; SIRI, systemic inflammatory response index.

* of Kruskal–Wallis test.

### 3.3 vHIT Findings and Correlation Analyses

Topographic classification of vestibular involvement based on vHIT demonstrated superior vestibular nerve involvement in 24 patients, inferior vestibular nerve involvement in 8 patients and combined involvement in 9 patients. The lowest canal gain recorded on vHIT was used as an exploratory indicator of vestibular dysfunction severity. Spearman correlation analysis showed no significant correlation between vestibular dysfunction severity and NLR, PLR, MLR, SII or SIRI (*p* = 0.893, *p* = 0.921, *p* = 0.239, *p* = 0.779 and *p* = 0.916, respectively). In addition, no significant correlation was found between length of hospitalisation and NLR, PLR, MLR, SII or SIRI (*p* = 0.602, *p* = 0.220, *p* = 0.934, *p* = 0.673 and *p* = 0.866, respectively).

### 3.4 Pairwise Post Hoc Comparisons

Table 3 presents the Bonferroni‐corrected *p* values for pairwise post hoc comparisons. Median values of NLR, SII and SIRI were significantly higher in the severe vestibular neuritis group compared to the control group (*p* = 0.032, *p* = 0.019 and *p* = 0.042, respectively). However, comparisons between the mild vestibular neuritis group and healthy controls revealed no significant differences in NLR, SII and SIRI (*p* = 0.197, *p* = 0.232 and *p* = 0.355, respectively), nor were there any differences between the mild and severe vestibular neuritis groups (*p* = 1).

**TABLE 3:** Bonferroni correction applied *p* values (p _adjusted_) of pairwise post hoc comparisons.

	NLR	SII	SIRI
Severe versus Control	**0.032**	**0.019**	**0.042**
Mild versus Control	0.197	0.232	0.355
Severe versus Mild	1	1	1

*Note:* Bold values indicate statistically significant adjusted *p* values (*p* < 0.05).

Abbreviations: NLR, neutrophil to lymphocyte ratio; SII, systemic immune‐inflammation index; SIRI, systemic inflammatory response index.

### 3.5 ROC Analysis

Figure 1 illustrates the ROC curve analysis, demonstrating the predictive value of NLR, SII and SIRI for severe vestibular neuritis. The receiver operating characteristic (ROC) analysis yielded the following AUC values: SII = 0.66 (95% CI, 0.526–0.795), SIRI = 0.645 (95% CI, 0.507–0.784) and NLR = 0.645 (95% CI, 0.504–0.787). The cutoff values indicating severe vestibular neuritis were 512.78 for SII (sensitivity: 70%, specificity: 55%), 1.11 for SIRI (sensitivity: 62%, specificity: 55%), and 2.07 for NLR (sensitivity: 70%, specificity: 60%).

**FIGURE 1:**
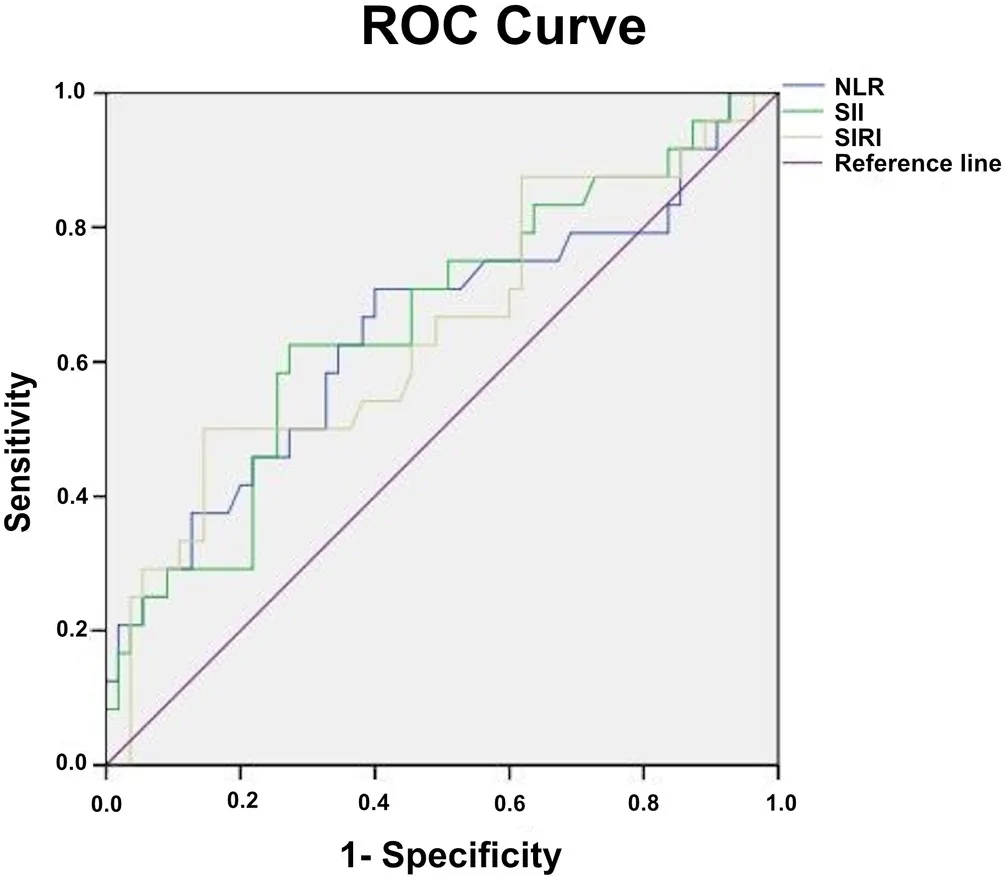
ROC curve analysis demonstrated the predictive value of NLR, SII and SIRI for severe vestibular neuritis.

## 4 Discussion

### 4.1 Principal Findings and Comparison With Previous Literature

Several hypotheses have been proposed to explain vestibular neuritis. The infectious hypothesis suggests that viral reactivation, particularly herpes simplex virus Type 1 (HSV‐1), may contribute to its development [13, 14]. This view is supported by the seasonal clustering of vestibular neuritis and autopsy studies showing HSV‐1 DNA in human vestibular ganglia, suggesting that latent viral infection and subsequent inflammation may damage the vestibular nerve.

A vascular mechanism has also been proposed. In some patients, elevated inflammatory mediators such as fibrinogen, TNF‐α and cell adhesion molecules support vascular inflammation and endothelial dysfunction [15]. This inflammatory response may promote microvascular compromise and ischaemia of vestibular structures, resulting in vestibular dysfunction [11]. Therefore, the elevated SII, SIRI and NLR values observed in the severe vestibular neuritis group may reflect not only a more severe clinical course and prolonged spontaneous nystagmus, but also mechanisms involved in disease pathogenesis.

Considering the previously reported link between vestibular neuritis and inflammatory as well as microvascular pathways, this study investigated whether inflammatory activity contributes to vestibular neuritis pathogenesis. In this context, the duration of spontaneous nystagmus—an objective clinical manifestation of vertigo—was regarded as a reliable indicator of disease severity, as prolonged nystagmus directly reflects impairment in daily functioning and overall well‐being. The study further examined whether the duration of spontaneous nystagmus is associated with systemic inflammatory biomarkers, including NLR, PLR, MLR, SII and SIRI.

Our findings indicate that inflammatory and microvascular mechanisms play a key role in vestibular neuritis, as evidenced by the significantly higher SII, SIRI and NLR values observed in patients with severe disease compared to the control group. This elevation reflects intensified systemic inflammation and greater vestibular dysfunction, supporting the notion that these mechanisms contribute to both the development and progression of the disorder. Given that disease severity in vestibular neuritis closely corresponds to impairment in daily functioning and overall well‐being, the observed relationship between elevated inflammatory indices and prolonged spontaneous nystagmus highlights their potential as predictive clinical markers of disease severity.

CBC is a routinely available tool for evaluating systemic inflammation. Elevated neutrophil and platelet counts reflect inflammatory activity, whereas decreased lymphocyte counts are associated with impaired health status [16]. NLR is a well‐established indicator of systemic inflammation, showing prognostic relevance for adverse clinical outcomes in various conditions, including cardiovascular, renal and malignant diseases or benign tumours like vestibular schwannoma [17, 18]. PLR has been linked to thrombosis risk, as platelets play a central role in thrombus formation and may increase ischemic complications like acute coronary syndrome [19]. An elevation in platelet volume may influence haemorrhage and contribute to inflammatory processes, thereby increasing the risk of ischemic complications [20, 21]. Both NLR and PLR have emerged as simple, cost‐effective biomarkers that reflect systemic inflammatory burden and thrombotic potential more reliably than isolated leukocyte or platelet parameters. Building upon this concept, our study extended beyond the conventional inflammatory markers addressed in the current literature and focused on novel composite indices—namely SII and SIRI—which incorporate platelet counts together with neutrophil, lymphocyte and monocyte levels, thereby providing a more integrated assessment of systemic inflammatory and thrombotic activity in vestibular neuritis.

Although the viral aetiology of vestibular neuritis has long been proposed, the evidence remains inconclusive. Viral infections are typically associated with a relative decline in neutrophil counts and an elevation in lymphocyte levels, which would be expected to result in a lower NLR. However, the elevated NLR values observed in vestibular neuritis suggest the involvement of additional mechanisms beyond a purely viral origin. Such elevations may indicate a systemic inflammatory and thrombotic state, supporting the ischemic hypothesis in disease pathogenesis. Recent studies have reported increased NLR values in vestibular neuritis, but did not address the relationship between NLR and disease severity [5]. To address this gap, our study evaluated not only NLR but also composite indices such as SII and SIRI, offering a broader perspective on systemic inflammatory and thrombotic mechanisms in vestibular neuritis.

SII, a relatively new biomarker, has demonstrated prognostic relevance across a wide range of malignancies, including gastrointestinal, pulmonary and genitourinary cancers. Beyond oncology, its clinical utility has also been highlighted in certain otologic disorders such as idiopathic sudden sensorineural hearing loss [8] and idiopathic facial paralysis [9]. SIRI was previously reported as a novel indicator for Bell's palsy [10]. To the best of our knowledge, no previous studies have specifically examined the association of SII and SIRI with vestibular neuritis, suggesting that the present study may contribute novel insights into this area.

### 4.2 Pathophysiological and Clinical Implications

Our results showed that SII, SIRI and NLR values were significantly higher in patients with severe vestibular neuritis compared with controls. These findings suggest an association between increased systemic inflammatory burden and a more severe clinical course. Although SII demonstrated the highest discriminative performance in ROC analysis (AUC = 0.66), the overall predictive ability of these indices was modest, and none was sufficient to reliably distinguish severe disease on its own. Nevertheless, these biomarkers may still reflect the degree of systemic inflammatory burden in vestibular neuritis. In the exploratory vHIT‐based analyses, superior vestibular nerve involvement was the most common topographic pattern. However, inflammatory biomarkers were not significantly correlated with the lowest canal gain on vHIT or with length of hospitalisation. This may suggest that, although elevated inflammatory indices are associated with the clinically severe group, they do not necessarily parallel canal‐specific vestibular dysfunction measured by vHIT, which is consistent with previous studies showing that vHIT gain does not always correlate with subjective dizziness severity or chronic symptom burden in vestibular neuritis [22, 23]. From a pathophysiological perspective, the observed elevations may support the view that inflammatory mechanisms contribute to symptom burden and disease expression in vestibular neuritis. Since previous studies have reported conflicting results on the effectiveness of anti‐inflammatory treatment in vestibular neuritis, our findings may help guide future research to determine whether patients with higher inflammatory burden are more likely to benefit from anti‐inflammatory treatments [24, 25].

### 4.3 Limitations

This study has several limitations, most of which arise from its retrospective design. First, the retrospective nature of the study precludes causal inference. Second, this was a single‐centre study, which may limit the generalisability of the findings to other clinical settings and patient populations. In addition, inflammatory markers were assessed only at presentation, and serial changes over time could not be evaluated. Finally, because of the retrospective design, long‐term follow‐up data regarding symptom duration, recovery course and persistence of vestibular dysfunction were not available in a standardised manner; and therefore the prognostic significance of elevated SII, SIRI and NLR remains uncertain. Future prospective studies with larger multicenter cohorts, serial biomarker assessments and standardised long‐term outcome measures are needed to clarify the diagnostic and prognostic relevance of these indices in vestibular neuritis.

## 5 Conclusion

NLR, SII and SIRI were elevated in patients with severe vestibular neuritis, suggesting an association between increased systemic inflammatory burden and a more severe clinical course. Among these markers, SII showed the highest discriminative performance, although the overall predictive value remained modest. These findings support the possibility that inflammatory mechanisms contribute to disease expression in vestibular neuritis. Further prospective studies are required to confirm these results and to determine the diagnostic, prognostic and potential therapeutic relevance of these biomarkers.

## Author Contributions

Protocol/project development: T.T., M.U.A., H.Ç., K.G.T., T.Y., G.K., H.Y. and Ö.H. Data collection or management: T.T., M.U.A., H.Ç., K.G.T., T.Y., G.K., H.Y. and Ö.H. Data analysis: T.T., M.U.A., H.Ç., K.G.T., T.Y., G.K., H.Y. and Ö.H. Project administration: T.T., M.U.A., H.Ç., K.G.T., T.Y., G.K., H.Y. and Ö.H. Manuscript writing/editing: T.T., M.U.A., H.Ç., K.G.T., T.Y., G.K., H.Y. and Ö.H.

## Funding

The authors have nothing to report.

## Ethics Statement

This study was conducted following approval from the Institutional Ethics Committee of Balıkesir University (IEC Number: 2023/112) and adhered to the principles of the Declaration of Helsinki.

## Conflicts of Interest

The authors declare no conflicts of interest.

## Data Availability

The data that support the findings of this study are available from the corresponding author, upon reasonable request.
